# Transforming Surgical Training: The Evolution of the COSECSA eLogbook Through a Theory of Change Framework

**DOI:** 10.1002/wjs.70573

**Published:** 2026-09-19

**Authors:** Lawa Shaban, Lucia Brocato, Eric O'Flynn, Abebe Bekele, Eric Borgstein, Laston Chikoya, Amani Pascal Uiso, James Geraghty, Juan Carlos Puyana

**Affiliations:** ^1^ Institute of Global Surgery RCSI Dublin Ireland; ^2^ College of Surgeons in East, Central and Southern Africa Arusha Tanzania; ^3^ Royal College of Surgeons in Ireland Dublin Ireland

**Keywords:** electronic logbook, global surgery, low‐resource settings, surgical training, theory of change, workforce development

## Abstract

**Introduction:**

Surgical training logbooks are vital for tracking trainee experience and training quality. As the College of Surgeons of East, Central and Southern Africa (COSECSA) expanded across 24 countries, its paper‐based system outgrew the capacity of paper logbooks. In partnership with the Royal College of Surgeons in Ireland, COSECSA developed an electronic logbook (eLogbook) to enhance data collection, trainee monitoring, and quality assessment. This study reviews its evolution using a theory of change framework to inform future surgical training needs in sub‐Saharan Africa and beyond.

**Methods:**

We applied a retrospective theory of change (ToC) framework to analyze the development, implementation, and impact of the COSECSA eLogbook. Data sources included archived project records, eLogbook data, and published articles utilizing logbook data. Descriptive summaries were generated to analyze the logged surgical procedures.

**Results:**

Phased rollout began in 2015 with full adoption in 2017. By the end of 2025, 1806 trainees had logged 712,118 procedures. The eLogbook facilitated training standardization across multiple countries and improved monitoring. Studies utilizing logbook data have identified disparities in procedural exposure, training gaps, and speciality‐specific challenges. The logbook also supported quality assurance and program improvement.

**Discussion:**

Key challenges include limited internet access, necessitating offline functionality, and initial resistance to digital adoption mitigated through training and iterative refinements. Lessons from COSECSA informed the development of a similar logbook for the College of Anesthesiologists in East, Central, and Southern Africa (CANECSA).

**Conclusion:**

The COSECSA eLogbook has revolutionized surgical training documentation, enabling data‐driven evaluation and standardization across programs. It showcases how digital tools and long‐term international collaboration can strengthen surgical education in low‐resource settings.

## Graphical abstract

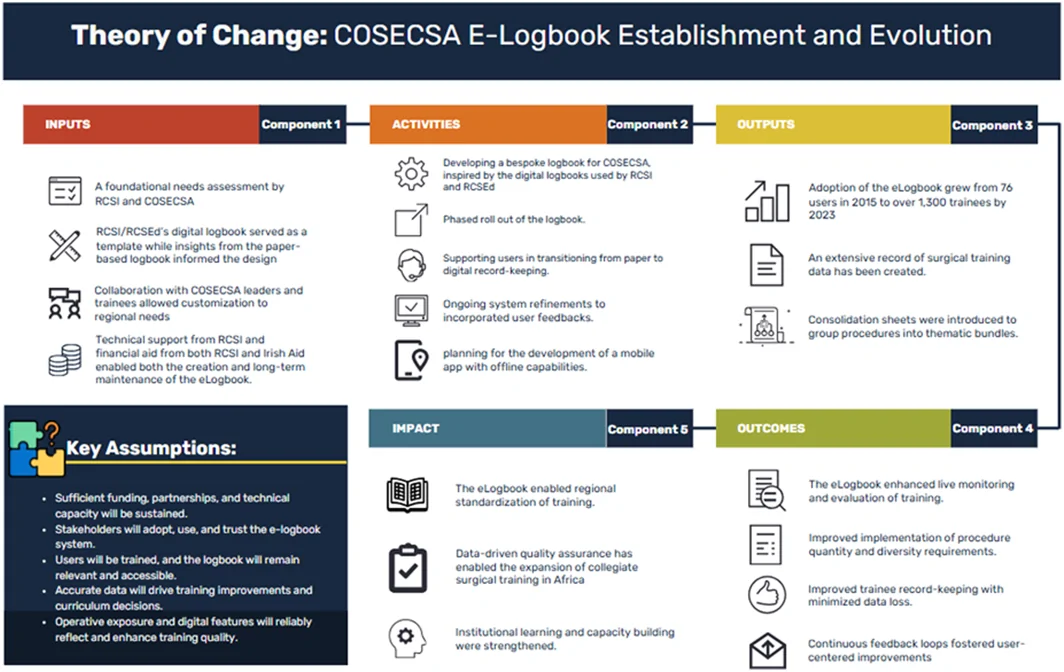

## 1 Introduction

Surgical training logbooks are widely regarded as essential tools in medical education, enabling trainees to document key aspects of their clinical experience, including patient demographics, procedures performed, operative roles, and surgical outcomes [1]. These records support reflective learning, facilitate the assessment of skill progression, and serve as critical data sources for evaluating training programs [2] and trainees' readiness for independent practice [3].

The College of Surgeons of East, Central, and Southern Africa (COSECSA) initiated its surgical training program in 2003, initially relying on paper‐based logbooks to monitor trainee progress [4]. These were used across a dispersed network that has since expanded to 155 training sites across 24 countries, covering an area more than twice the size of the European Union [5]. As trainee numbers grew, the limitations of paper‐based systems became apparent: by 2013, the system struggled to handle the increasing volume of data, and logbook review had become unwieldy and inefficient, challenges compounded by COSECSA's extensive geographical spread.

COSECSA has partnered with the Royal College of Surgeons in Ireland (RCSI) since 2007 in a “whole college” collaboration supported by Irish Aid [6]. In 2014, the two institutions conducted a comprehensive needs assessment that identified key areas in which a digital platform could enhance documentation, streamline data collection, and improve the monitoring of trainee progress. Accordingly, they collaboratively developed an eLogbook to provide a scalable, accessible, and analyzable platform for recording surgical exposure, capturing 16 variables for each procedure (Table 1).

**TABLE 1:** Fields recorded for surgical logbook entries.

Fields recorded	Options for the field
Country	Any
Hospital	Name of all COSECSA‐accredited hospitals and one option for unaccredited hospitals
Date of operation	Selected from a calendar
Patient ID	Optional
Patient age	In both years and months
Patient sex	Male; Female
Role	Performed—unsupervised; performed; assisted
Level of help	No help; show and tell; active help; passive help; supervision only
Supervised by	Name of supervisor
Specialty	General surgery; orthopedic surgery; urology; pediatric surgery; plastic surgery; otorhinolaryngology; neurosurgery; cardiothoracic surgery
Operation type	A curated list of 3048 operations
Urgency type	Emergency; elective
Outcome	Discharged improved; discharged against advice; deteriorated; death
ASA^a^ classification	ASA^a^ 1 through ASA^a^ 6
Intraoperative complication (classintra classification)	Grade 0 to grade 5
Notes	Optional

^a^ American Society of Anesthesiologists Physical Status Classification System.

As the surgical workforce must expand to ensure safe global access to surgery and anesthesia, training programs must verify competence, protect patients, and plan workforce needs despite limited resources. Reliable operative records are essential, as case volume and diversity correlate with proficiency [7]. The American Board of Surgery, for example, recently raised its minimum case requirement from 750 to 850 [8] and defined learning curves that specify the caseload needed to reach proficiency [9]; without accurate logs, educators cannot detect when these thresholds are missed. In East, Central, and Southern Africa (ECSA), the surgical workforce averages just 0.6 surgeons per 100,000 far below the Lancet Commission's benchmark of 20, and tracking surgical volume is a key metric in global surgery [10, 11]. Without robust data, gaps in training and service delivery cannot be effectively addressed.

This paper documents the development and evolution of the COSECSA eLogbook, examining its phased rollout, subsequent improvements, challenges encountered, and long‐term impact on surgical training. It also serves as a case study for implementing electronic data capture in medical training within low‐resource settings, demonstrating how digital tools can strengthen the training environment and yield actionable insights into trainee performance.

## 2 Methods

### 2.1 Study Design and Rationale

This study employed a retrospective theory of change (ToC) framework to analyze the collaborative development, phased implementation, and evolution of the COSECSA eLogbook within the RCSI–COSECSA partnership. By linking inputs and activities to outcomes through explicit assumptions (Figure 1), the ToC clarifies how and why specific actions produce results and helps define future goals as workforce demands grow [12, 13, 14]. This approach enabled a clear reconstruction of the development process and identification of likely causal pathways.

**FIGURE 1:**
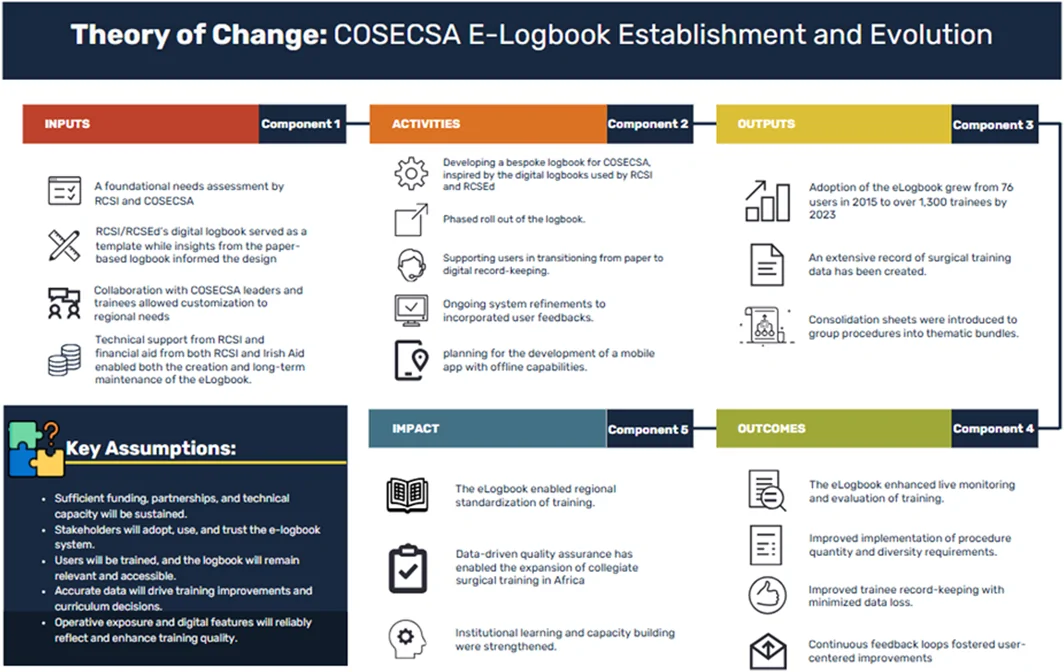
Theory of change: Cosecsa E‐logbook development, evolution, and impact.

### 2.2 Data Sources

Multiple data sources were triangulated: (i) archived project documentation; (ii) published eLogbook usage data [15]; and (iii) peer‐reviewed publications based on eLogbook data [16, 17, 18, 19, 20]. These provided complementary perspectives on design decisions, stakeholder contributions, implementation adaptations, and contextual influences.

### 2.3 Data Extraction and Descriptive Summary

A decade of historical eLogbook data was used to generate summary metrics describing the volume and characteristics of recorded procedures, including urgency, operative role, level of help, speciality, and procedure type as defined by the inherited RCSI/RCSEd procedure list [15]. Descriptive summaries and visualizations were produced in Microsoft Excel, following basic validation checks for completeness and internal consistency.

### 2.4 Retrospective Development of TOC Model

The analysis followed four stages: (1) collation of relevant documents, usage data, and published analyses; (2) timeline development, with two reviewers identifying and ordering key events; (3) mapping events into ToC domains (Inputs to Impacts) and linking them through causal pathways and assumptions; and (4) stakeholder validation with multiple manuscript review rounds by COSECSA and RCSI.

### 2.5 Causal Contribution Inference

Inference drew on process‐tracing logic: temporal precedence (activities systematically preceding observed outputs) and pattern alignment between hypothesized intermediate changes and empirical utilization metrics.

### 2.6 Bias Mitigation Strategies

To minimize bias, data were triangulated across sources, prioritizing written records over retrospective accounts. The analysis spanned the full program lifecycle (2014–2025) to avoid selective reporting. Two investigators independently reviewed and reconciled the timeline, and draft findings were externally validated by COSECSA and RCSI stakeholders.

### 2.7 Reporting Standards and Ethical Considerations

Methods and reporting adhered to SQUIRE 2.0 quality improvement guidelines [21]. The data presented are either publicly available or constitute nonsensitive educational research data; learners are not identifiable, and the research poses minimal risk to them. The COSECSA Institutional Review Board assessed the research and confirmed that ethical review was not required and issued a waiver (Ref. No. COSECSA/REC/2025/10).

### 2.8 Declaration of AI Usage

AI use in this manuscript is reported in line with the TITAN 2025 guidelines [22]. AI use was limited to language checking with Grammarly; no generative AI was used. The authors take full responsibility for the manuscript's content and accuracy.

## 3 Results

We identified key steps, decisions, and likely causal pathways underpinning successful deployment and adoption. The ToC framework comprises five components: Inputs, Activities, Outputs, Outcomes, and Impact, each capturing crucial aspects of the process.

### 3.1 Inputs

A 2014 needs assessment conducted by RCSI and COSECSA identified substantive limitations in the paper‐based logbook system, including data loss, inadequate tracking of surgical exposure, and the absence of centralized oversight. Trainees calculated procedure totals manually, and logbooks were reviewed only immediately before examinations, precluding timely analysis. These deficiencies indicated a clear requirement for digitization, predicated on the assumption that an electronic system would enhance data accuracy, accessibility, and oversight, thereby strengthening supervision and evaluation. To ensure continuity and minimize development costs, the eLogbook retained core elements of the paper format and drew structurally on the RCSI and Royal College of Surgeons of Edinburgh (RCSEd) logbooks. RCSI provided the requisite technical and financial support.

### 3.2 Activities

Development, rollout, and refinement encompassed: review of comparator digital logbooks, a phased rollout, structured training for users and administrators, iterative system refinements, and the subsequent release of a mobile application with offline functionality.

A bespoke logbook was developed following the needs assessment, integrating key fields from the paper‐based instrument into a digital platform. Iterative consultation with COSECSA leadership, trainers, and trainees ensured contextual relevance and responsiveness to user feedback. The resulting eLogbook comprised 14 mandatory and two optional fields covering patient demographics, procedure type, trainee role, level of help, supervision, and outcomes, as shown in Table 1. The procedure list was directly adopted from the existing RCSI/RCSEd logbook, subsequently modifying it over successive years to reflect local practice and user demand. The list is not mapped to any recognized procedural classification system. These activities rested on the assumption that stakeholder engagement and structured design were prerequisites for adoption and usability.

The phased rollout commenced in 2015, initially enrolling new trainees only, to safeguard data quality and avoid retrospective entry burdens; by 2017, coverage extended to most trainees. Early feedback informed enhancements, including automated “consolidation sheets” that grouped procedures into thematic bundles with target volumes, enabling assessment of operative experience against program expectations. Once mandatory, use was enforced through examination eligibility criteria.

Subsequent refinements narrowed the range of selectable procedures and operative roles, introduced a level of help field, assigned complexity scores to each procedure, and introduced an “intraoperative complications” field to capture immediate outcomes alongside the existing “Outcomes” field, which records longer‐term postoperative morbidity and mortality. Routine reports afforded supervisors and program directors enhanced access to individual and aggregate trainee data.

Continuous user feedback guided further iteration: obsolete procedures were retired and the interface streamlined to reduce barriers to use. In 2024, to mitigate connectivity constraints, development of a mobile application with offline functionality was initiated and subsequently released in 2025 to reduce the interval between case performance and documentation.

### 3.3 Outputs

The outputs included widespread adoption and extensive data collection, yielding a comprehensive dataset that offered valuable insights into the quality and scope of surgical training across the COSECSA region. Uptake grew markedly, from 76 trainees in 2015 to 1806 by the end of 2025, who collectively recorded 712,118 surgical operations.

The COSECSA eLogbook has supported multiple studies on surgical training in ECSA. Research on operative volumes has revealed gaps in general surgery training and disparities in case exposure across specialities and countries [16]. Studies on pediatric surgical training emphasize the need for post‐certification training due to limited exposure to subspecialties [17]. Other studies have examined the sustainability of electronic logbooks, highlighting challenges such as limited internet access and the need for context‐specific solutions in low‐ and middle‐income countries [18]. Exam performance analysis revealed that factors such as language proficiency and institutional affiliation impact surgical trainees' outcomes [19]. Additionally, research on laparoscopy use in the region showed limited adoption with significant variation by income level and hospital type, highlighting a greater need for exposure to minimally invasive techniques [20]. Also, using COSECSA logbook data, Parker et al. found that faith‐based hospitals in ECSA had significantly lower perioperative mortality rates than public and private hospitals [23]. These studies highlight the eLogbook's value in assessing COSECSA's training quality and identifying gaps in both training and infrastructure. However, their validity depends on the assumption that logbook data are accurate, complete, and truly reflect clinical practice.

The logbook data show that 65% of patients were male and 35% female. Elective procedures made up 64% of cases, whereas 36% were emergencies. Geographically, the highest volume of operations was recorded in Kenya (32%) followed by Uganda (12%) and Zambia (10%). Overall, 99% of procedures occurred in COSECSA member or partner countries with just under 1% in other African nations and outside Africa Figure 2.

**FIGURE 2:**
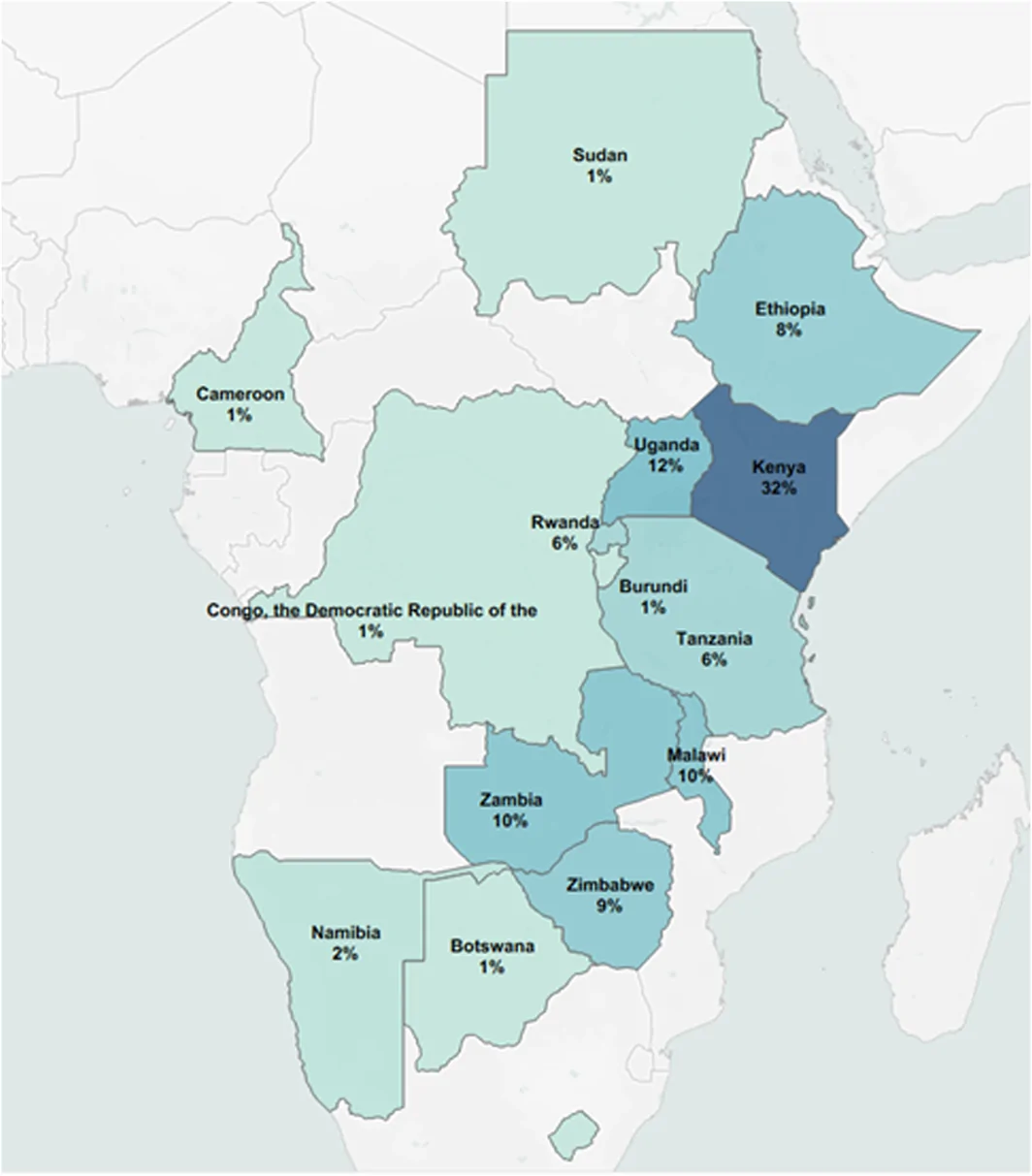
Geographical distribution of COSECSA logged.

By speciality, general surgery accounted for 37% of cases, followed by orthopedics (25%), pediatrics (10%), urology (9%), plastic surgery (9%), neurosurgery (7%), otorhinolaryngology, and cardiothoracic surgery (1%). The most common procedures included hernia repair, femur fracture fixation, transurethral prostatectomy, skin grafting, craniectomy for subdural collection, adenotonsillectomy, and feeding jejunostomy/gastrostomy insertion (Table 2). The largest patient age group was 19–39 years Figure 3.

**TABLE 2:** Distribution of specialities and frequency of most common procedures logged in the COSECSA E‐logbook.

Speciality (% of total operations)	Three most common operations	% in the speciality
General surgery (37%^a^)	Hernia inguinal—adult	7%
	Exploratory laparotomy (no other procedure)	6%
	Appendicectomy (all types)	6%
Orthopedic surgery (25%^a^)	Diaphyseal femur fracture intramedullary nailing	8%
	Diaphyseal tibial fracture intramedullary nailing	3%
	Supracondylar elbow fracture MUA +/− percutaneous wires	3%
Pediatric surgery (10%^a^)	Hernia inguinal—pediatric	13%
	Colostomy—formation of end/loop colostomy/ileostomy/Double‐barrel colostomy	5%
	Repair of umbilical hernia	5%
Urology (9%^a^)	Transurethral prostatectomy—TURP—(any)	9%
	Open prostatectomy—(any)	6%
	Urethroplasty (stage/complete)	5%
Plastic surgery (9%^a^)	Skin graft	14%
	Primary repair unilateral cleft lip	2%
	Release of burn scar and Full‐thickness graft	2%
Neurosurgery (7%^a^)	Burr hole/craniectomy for subdural collection (incl. Csdh)	14%
	Insertion of a ventriculoperitoneal shunt	12%
	Craniotomy for extradural hematoma	9%
Otorhinolaryngology (2%^a^)	Adenotonsillectomy	13%
	Open tracheostomy	7%
	Tonsillectomy	6%

^a^ Totals may not sum to 100% due to rounding.

**FIGURE 3:**
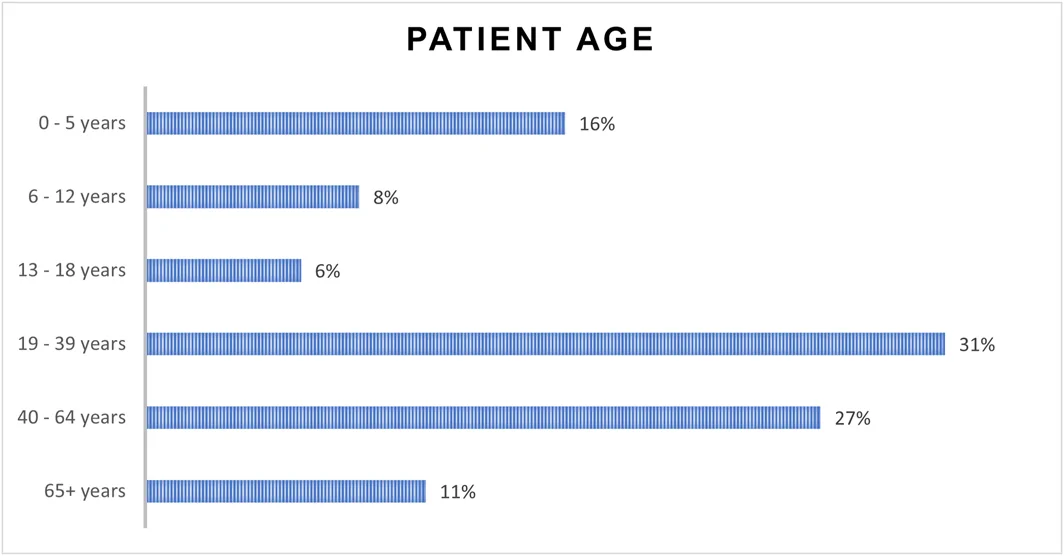
Age distribution of patients in the COSECSA surgical training E‐logbook.

### 3.4 Outcomes

The eLogbook enhanced training monitoring and evaluation by enabling real‐time tracking of case breadth and complexity. Consolidation sheets and complexity metrics afforded trainers a clear overview of both volume and variety, supporting evidence‐informed review and curricular adjustment. The transition from paper to digital records mitigated data loss and simplified tracking of experiences, facilitating certification and assessment. Ongoing feedback loops between trainees, trainers, and developers continue to drive iterative system improvement. These benefits rest on the assumption that recorded data reflect actual competence and progression, and that sustained use will inform curriculum development, policy, and systemic reform. Both COSECSA and RCSI have derived reciprocal insights with cross‐fertilization between their respective logbooks; the COSECSA Secretariat now leads administration and ongoing development.

### 3.5 Impact

The eLogbook has exerted a substantial long‐term impact on surgical training across the COSECSA region. A principal outcome is the standardization of training, enabling consistent logbook practices across multiple ECSA countries and ensuring uniformity in documenting and evaluating surgical exposure, assumed to contribute to more equitable training outcomes.

The eLogbook further supports data‐driven quality assurance through reliable records of trainee activity, informing programmatic improvements and demonstrating accountability to regulators, funders, and partners. It has also underpinned the expansion of surgical training in Africa: COSECSA is now the largest contributor to the ECSA surgical workforce. Although trainee growth cannot be attributed solely to the logbook, its integration has plausibly been instrumental in enabling and sustaining this expansion (Figure 4).

**FIGURE 4:**
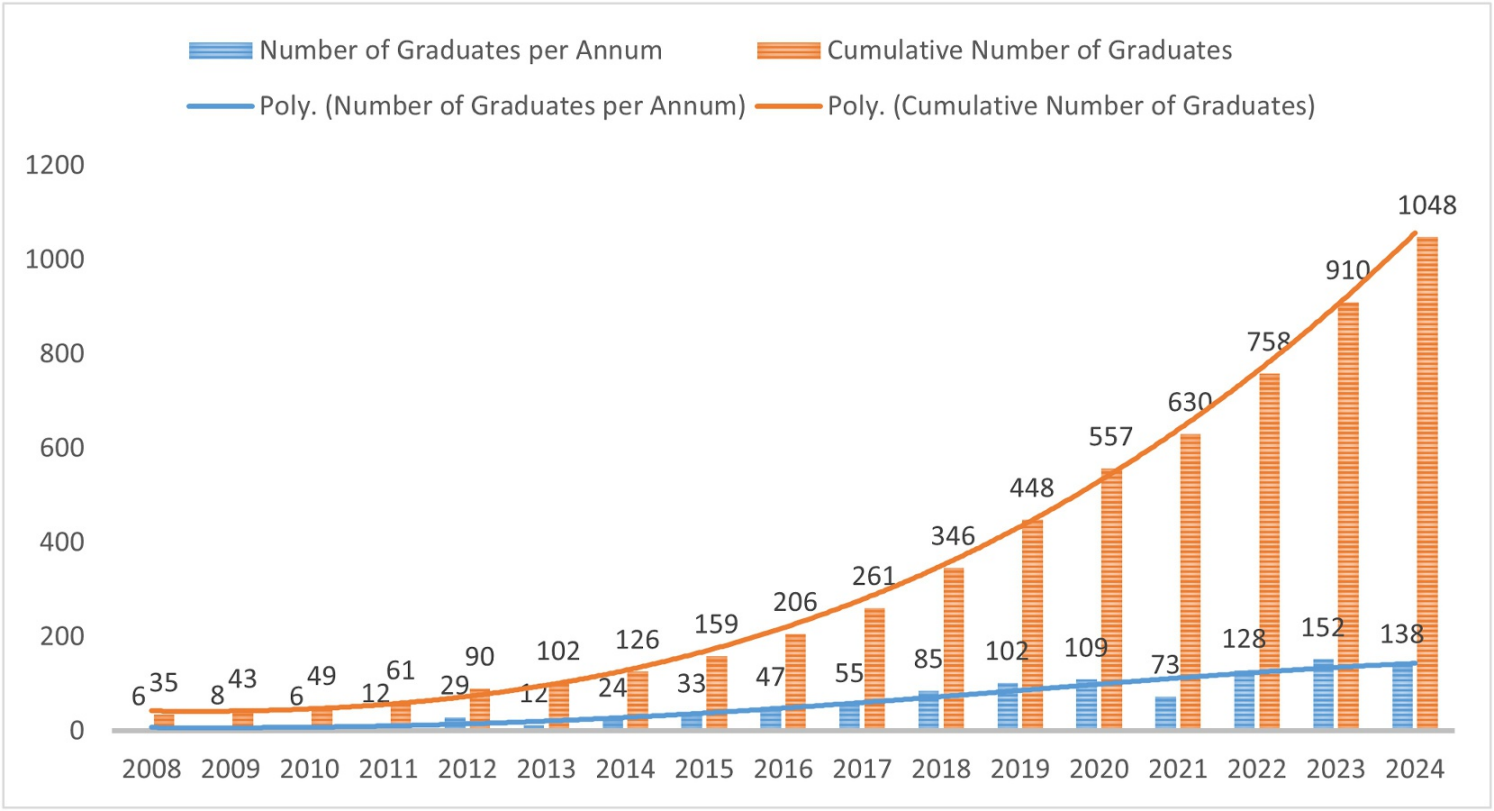
Annual and cumulative COSECSA graduates (2008–2024), showing exponential growth in the surgical workforce.

Finally, the retrospective analysis identified institutional learning and capacity building as central impacts; the eLogbook has demonstrated adaptability to evolving surgical practices, training requirements, and user feedback, fostering a sustainable training infrastructure. Quality assurance across geographically dispersed training sites and its demonstration to regulators, funders, and other stakeholders would not have been feasible without such an instrument. Arguably, a large decentralized training college could not have attained such success in its absence [6].

### 3.6 Assumptions and Contextual Factors

The successful implementation of the eLogbook was underpinned by several assumptions. The central belief was that digitization would mitigate issues such as data loss, limited accessibility, and limited oversight by improving data accuracy, enabling real‐time monitoring, and increasing engagement among trainers and examiners. This relied on sustained funding, strong partnerships, and technical capacity to design and maintain the platform.

Another assumption was that regional factors, such as internet access and digital literacy, would be sufficient to support consistent use. Adoption further depended on the system's user‐friendliness and the availability of adequate training resources. Stakeholder engagement during development was expected to strengthen ownership and uptake.

Other assumptions included that logged data would be accurate, complete, and representative; supervisors and institutions would engage meaningfully with the data; the volume and complexity of procedures could reliably indicate surgical competence. The system was expected to support evidence‐based improvements, equitable trainee assessment, and regional standardization.

Finally, it was assumed that institutional learning would be ongoing, with the system adapting to evolving training needs and user feedback. Scalability and sustainability depended on continued technical enhancements, such as offline functionality and institutional collaboration. These assumptions, alongside enabling contextual factors, were critical to the logbook's uptake and long‐term impact.

## 4 Discussion

By transitioning from a paper‐based system to a digital platform, COSECSA has successfully streamlined trainee record‐keeping and significantly improved data accessibility and quality assurance across multiple countries. The ToC framework has facilitated an in‐depth analysis, mapping the critical steps from foundational inputs, such as the initial needs assessment, through long‐term impacts, such as standardization of training and institutional learning. Notably, widespread user adoption and regional standardization underscore the effectiveness of the phased rollout approach, which has allowed incremental improvements based on real‐time feedback. The successful adoption of the logbook was demonstrated by Harrington et al. (2020), showing nearly 60% of trainees record a majority of their cases, whereas only around 10% rarely or never record their cases [18].

This project has generated significant learning for both colleges and has led to the development of a bespoke logbook for the College of Anesthesiologists in East, Central, and Southern Africa (CANECSA). This logbook was launched in January 2024 and contains over 20,000 records as of 31st October 2024. Furthermore, the COSECSA eLogbook shows that a lean, web‐based system, mandated by a training college and refined with user feedback, can standardize training in low‐resource settings. Its rapid adaptation for CANECSA highlights its portability. Successful replication depends on three factors: offline access, mandatory use enforced by a professional body with trainer access to real‐time data, and ongoing user‐driven improvements. With these in place, the model can be adopted elsewhere at minimal cost.

To our knowledge, this is the first detailed retrospective analysis of how a regionally mandated eLogbook was developed and institutionalized across 24 sub‐Saharan African countries. Using a ToC framework, it highlights how digital tools can support training standardization, program evaluation, and quality assurance at scale in complex, low‐resource settings.

### 4.1 Challenges and Lessons Learned

Technical barriers, particularly limited internet connectivity, hindered full adoption of the system in regions with underdeveloped digital infrastructure. The absence of offline functionality, frequently requested by users, made consistent digital record‐keeping difficult for trainees in low‐connectivity areas, prompting the development of an offline‐capable smartphone application released in early 2025.

Resistance from users accustomed to paper‐based record‐keeping presented a further obstacle. The project team addressed this through an iterative approach combining intensive training with the gradual introduction of support materials. This phased strategy proved essential for reducing resistance, promoting engagement, and ensuring successful integration, and the resulting insights will inform future enhancements of the eLogbook system. Another challenge is the inconsistent logging of procedures by trainees, a well‐documented issue across many regions and specialities, as noted in several studies on surgical education [24, 25].

Adapting the procedure list from the existing RCSI/RCSEd logbook, intended as a time‐saving measure, proved a misstep: its broad scope had limited relevance to COSECSA's training needs. Extensive iterative refinement was therefore required to remove redundancies and tailor the list to the regional surgical context. In hindsight, a bespoke list grounded in an established coding system should have been developed from the outset. Stakeholder feedback was instrumental in shaping the final product, a leaner, regionally relevant, and user‐friendly logbook that effectively supports COSECSA's training, research, and program evaluation.

Finally, the ToC will continue to guide decision‐making by providing a structured framework for anticipating outcomes, identifying necessary inputs, and assessing alignment with intended impacts. As the logbook evolves, key assumptions, including user engagement, data accuracy, and institutional uptake must be regularly reviewed and validated through research and stakeholder feedback.

### 4.2 Limitations

The retrospective ToC approach necessarily has some limitations: reconstructing causal pathways from archival documents and stakeholder recall may omit undocumented decisions, obscure timelines, and cannot establish a counterfactual, so links remain likely rather than proven. Second, the eLogbook dataset is self‐reported, exposing it to under‐recording of emergencies or low‐complexity cases when connectivity or time is scarce and to possible overstatement or miscoding of procedures where trainees aim to meet case thresholds. Consequently, both the causal map and the procedural volumes should be interpreted as indicative not definitive.

## 5 Conclusions

The COSECSA eLogbook project successfully transitioned surgical training documentation in ECSA from a cumbersome paper‐based system to a comprehensive digital platform. It demonstrates the transformative potential of digital tools in medical training in low‐resource settings, evidenced by the subsequent adoption of eLogbook by CANECSA, and exemplifies the impact of sustained, capacity‐building partnerships between aligned institutions committed to advancing surgical education. The introduction of an offline application further enhances usability and data completeness.

This study offers key lessons for global health and education initiatives, underscoring the value of phased implementation aligned with institutional policy, stakeholder‐driven system refinement, reliable usage data for monitoring and evaluation, and the portability of digital tools when core enablers are in place. The COSECSA experience illustrates how collaborative, context‐sensitive innovation can yield scalable and sustainable solutions in global surgical education.

## Author Contributions

**Lawa Shaban:** conceptualization, data curation, methodology, formal analysis, project administration, visualization, writing–original draft. **Lucia Brocato:** writing–review and editing, project administration, supervision. **Eric O'Flynn:** writing–review and editing, writing–original draft, conceptualization. **Abebe Bekele:** writing–review and editing. **Eric Borgstein:** writing–review and editing. **Laston Chikoya:** writing–review and editing. **Amani Pascal Uiso:** writing–review and editing, data curation, resources, software. **James Geraghty:** writing–review and editing. **Juan Carlos Puyana:** writing–review and editing, conceptualization, formal analysis, investigation, methodology, writing–original draft, project administration.

## Funding

Irish Aid supported the development of the logbook through the COSECSA–RCSI Collaboration Program; no direct funding was received for this manuscript.

## Ethics Statement

The COSECSA Institutional Review Board confirmed that ethical review was not required and issued a waiver (Ref. No. COSECSA/REC/2025/10).

## Conflicts of Interest

The authors declare no conflicts of interest.

## Data Availability

The data that support the findings of this study are available from the corresponding author upon reasonable request.
